# Deep learning enhanced the diagnostic merit of serum glycome for multiple cancers

**DOI:** 10.1016/j.isci.2023.108715

**Published:** 2023-12-13

**Authors:** Haobo Zhang, Si Liu, Yi Wang, Hanhui Huang, Lukang Sun, Youyuan Yuan, Liming Cheng, Xin Liu, Kang Ning

**Affiliations:** 1 Key Laboratory of Molecular Biophysics of the Ministry of Education, Hubei Key Laboratory of Bioinformatics and Molecular-imaging, Center of AI Biology, Department of Bioinformatics and Systems Biology, College of Life Science and Technology, Huazhong University of Science and Technology, Wuhan, Hubei, China; 2 Department of Epidemiology and Health Statistics, School of Public Health, Fujian Medical University, Fuzhou, Fujian, China; 3 Department of Laboratory Medicine, Tongji Hospital, Tongji Medical College, Huazhong University of Science and Technology, Wuhan, Hubei, China

**Keywords:** Cancer, Glycomics, Machine learning

## Abstract

Protein glycosylation is associated with the pathogenesis of various cancers. The utilization of certain glycans in cancer diagnosis models holds promise, yet their accuracy is not always guaranteed. Here, we investigated the utility of deep learning techniques, specifically random forests combined with transfer learning, in enhancing serum glycome’s discriminative power for cancer diagnosis (including ovarian cancer, non-small cell lung cancer, gastric cancer, and esophageal cancer). We started with ovarian cancer and demonstrated that transfer learning can achieve superior performance in data-disadvantaged cohorts (AUROC >0.9), outperforming the approach of PLS-DA. We identified a serum glycan-biomarker panel including 18 serum N-glycans and 4 glycan derived traits, most of which were featured with sialylation. Furthermore, we validated advantage of the transfer learning scheme across other cancer groups. These findings highlighted the superiority of transfer learning in improving the performance of glycans-based cancer diagnosis model and identifying cancer biomarkers, providing a new high-fidelity cancer diagnosis venue.

## Highlights

• Serum glycome is promising biomarkers for cancer diagnosis
• Deep learning improved the performance of glycome-based cancer diagnosis model
• Deep learning model identified potential serum glycan-biomarker panel for cancers
• Glycan derived traits sialylation is an important biomarker for ovarian cancer

## Graphical abstract

**Figure:**
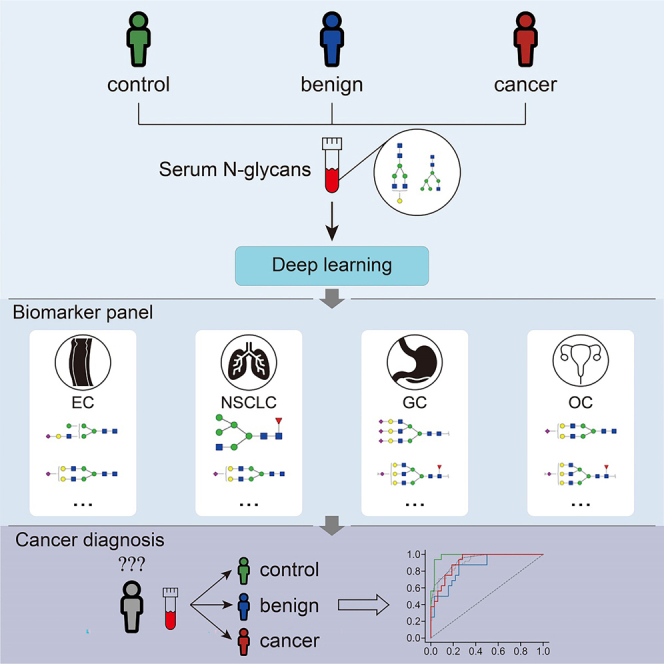

## Introduction

Cancer is a major public health problem worldwide and has become one of the most common causes of death.^1^^,^^2^ It can occur in various parts of the body and ultimately lead to serious consequences. For example, lung cancer, specifically non-small cell lung cancer (NSCLC), is the primary cause of respiratory system cancer-related deaths, accounting for 1.8 million deaths in 2020.^3^ Digestive system tumors, such as esophageal cancer (EC), gastric cancer (GC), colorectal cancer (CRC), and hepatocellular carcinoma (HCC) have high morbidity and mortality rates, making them prominent among malignant tumors.^4^ For reproductive system, prostate cancer and ovarian cancer are the most common cancer type in male and female, respectively.^5^^,^^6^^,^^7^ Notably, ovarian cancer has the highest mortality rate among all gynecological malignancies.^6^ Since these various cancers correspond to multiple systems, tumor biomarkers from multi-omics have also been examined.^8^^,^^9^^,^^10^^,^^11^^,^^12^ Most biomarkers focus on the underlying mechanisms of cancer development, such as gene mutations or abnormal expression. However, their effectiveness is not always fairly acceptable due to the complex relationship between genes and phenotypes. Additionally, conventional diagnostics relying on proteomic/genomic biomarkers are limited considering throughput, analysis speed, and invasiveness of sampling.^13^ It is urgently needed for novel approaches in cancer diagnosis, risk prediction, and treatment.

Glycans, closely involved in the downstream cellular metabolism, can be a promising source for the discovery of new reliable tumor biomarkers, which are more distal over genomic and proteomic approaches for precision cancer diagnostics.

Glycosylation is one of the most prevalent post-translational modifications and involves in many fundamental molecular and cell biology processes occurring in cancer, including tumor cell dissociation and invasion, tumor cell–matrix interactions, cell signaling transduction pathways, inflammation and immune response.^14^^,^^15^ It was reported that protein glycosylation was associated with the pathogenesis of many cancers, such as gastric cancer, colorectal cancer, and hepatocellular carcinoma.^14^^,^^16^^,^^17^^,^^18^^,^^19^^,^^20^ Particularly, glycans play important role in immunosurveillance, with most of the key factors involved in immune response are glycosylated.^15^^,^^21^^,^^22^^,^^23^ Previous studies demonstrated that antitumor immunity was an important barrier to tumor formation and progression, while evading immune destruction have been listed as an emerging hallmark of cancer.^24^ These findings linked the glycosylation with cancers, and the cancer states may be reflected in the appearance of abnormal glycans or altered quantitative proportions within the glycome.^7^^,^^14^^,^^25^ The important role of glycans in the development and progression of cancer suggests that changes in serum glycosylation were promising cancer biomarkers, which can aid in the precision screening and diagnosis of the cancer disease,^15^^,^^21^ as well as improve the survival rate and reduce the patient suffers or economic burden. Moreover, serum-glycan-based markers demonstrate superiority to traditional biopsy and computed tomography methods, because serum analysis is non-invasive and low-cost for point-of-care testing (POCT).^13^

There are existing data analysis methods for glycome biomarkers identifying, such as differential analyses including T-test and Wilcoxon-Mann-Whitney U test for the diagnosis of prostate cancer, which have found that the N-glycopeptide IgG2-GP09 (EEQFNSTFR (H5N5S1)) was dramatically elevated in patients with prostate cancer.^26^^,^^27^ In another study by Ruhaak *at al.*, partial least squares discriminant analysis (PLS-DA) was conducted to assess the potential of glycosylation profiles for the use of diagnosis of ovarian cancer.^28^ However, the differential analyses were primarily directed toward the examination of individual glycans in isolation, whereas PLS-DA involved a straightforward linear amalgamation of diverse glycan characteristics for cancer diagnosis. Therefore, these traditional methods were often inadequate in characterizing the relationship between glycans and cancer, which hindered them from more accurate and pervasive cancer diagnosis, as well as the identification of promising biomarkers.

The development of artificial intelligence provides a new and powerful approach for health care, including rapid and accurate image interpretation, potential cancer biomarkers identification, and effective early diagnosis.^29^^,^^30^^,^^31^^,^^32^ Recent studies on the relationship between glycosylation and cancer diseases have utilized machine learning models to assist in the classification between patients with cancer and healthy individuals, as well as to screen for relevant glycan biomarkers.^33^ Since the training data are the knowledge source of the machine learning models, it could be difficult to build models with decent performance for groups with small sample size. On the other hand, differences among datasets could potentially form challenges in maintaining comparable performance of a model trained on one cohort when applied to another cohort. These facts may have profound negative impacts on health care for the data-disadvantaged groups and generate new health care disparities.^34^^,^^35^^,^^36^

In this study, we have investigated the utility of deep learning techniques in the discrimination power of glycome for multiple cancer diagnosis using the consecutive data from our previous studies (Unpublished). We have found that transfer learning scheme, in many cases, can enhance the performance of machine learning models for groups that have limited data. We focused on the ovarian cancer (OC) and investigated the role of transfer learning method in improving the performance of the serum N-glycome based model in data-disadvantaged cohorts. Our study also identified several Glycosylation biomarkers for ovarian cancer, some of which were specific to the data-disadvantaged cohorts. Finally, we conducted the experiments on other cancer groups including non-small cell lung cancer (NSCLC), gastric cancer (GC) and esophageal cancer (EC) to validate the universality of the transfer learning scheme. These results provided a perspective for an unbiased machine learning paradigm, which is essential for reducing health care disparities arising from the data inequality.

## Results

### Sample cohort and experimental design for ovarian cancer group

**Figure 1:**
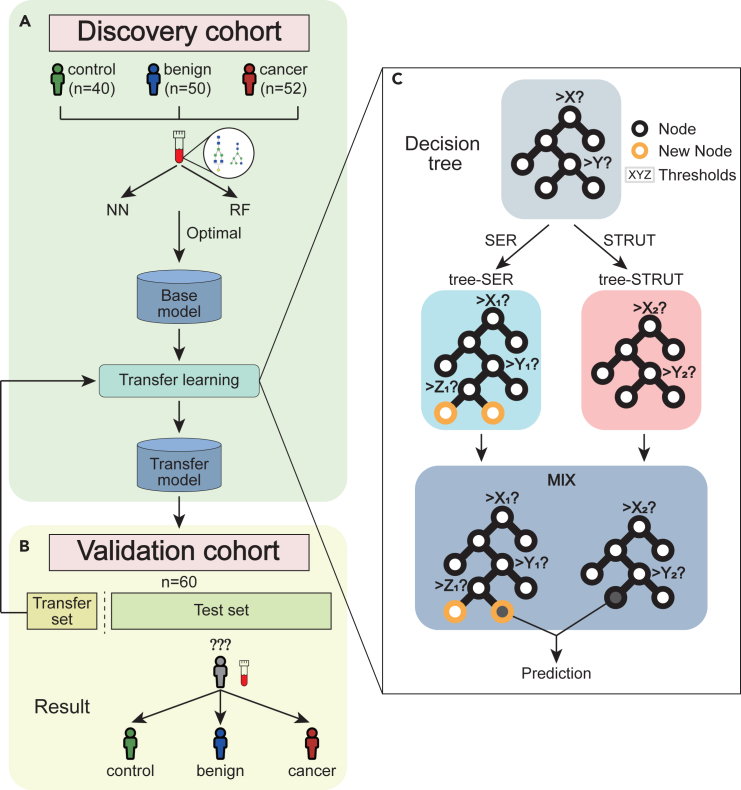
Framework for N-glycome based ovarian cancer diagnostic model and transfer learning (A) 142 subjects including 40 healthy controls, 50 benign neoplasm and 52 patients with cancer from discovery cohort were recruited to establish the diagnostic model based on the N-glycome. (B) A small proportion of the validation cohort (transfer set) was used to generate the transfer model and the remaining data (test set) was used for model evaluation. (C) Corresponding transfer models were built by SER and STRUT algorithms respectively: SER seeks to obtain local optimal modifications of decision tree, while STRUT modifies only the decision nodes thresholds without altering the structure. The resulting forests were combined to obtain a final random forest transfer model. SER, Structure Expansion Reduction; STRUT, Structure Transfer.

Serum samples including N-glycans and glycan derived traits were taken from 142 participants, including 40 healthy controls, 50 patients with benign neoplasm and 52 patients with ovarian cancer (Figure 1A, discovery cohort, Table 1). Based on the subjects from discovery cohort, neural network (NN) and random forests (RF) models were trained and compared to obtain the optimal model defined as base model. A new transfer model was developed from base model using transfer learning algorithms consisted of SER and STRUT algorithms (Figure 1B). Finally, the transfer model was tested in the validation cohort including 60 participants and identified the potential serum glycans biomarkers (Figure 1C, validation cohort, Table 1).

**Table 1:** Demographic and clinical characteristics of study participants

Characteristics	Control	Benign	Cancer	P.values
**Discovery cohort**				

**Ovarian cancer**	N = 40	N = 50	N = 52	/
Age (years), median (range)	54 (26–69)	51.5 (18–80)	52.5 (19–75)	0.36
Female sex, n (%)	40 (100.0)	50 (100.0)	52 (100.0)	/
Ethnicity (n)	Chinese (40)	Chinese (50)	Chinese (52)	/
Pausimenia, n (%)	/	33 (66.0)	32 (61.5)	0.64
Clinical stage (Early, Advanced)	/	/	18, 34	/
**Non-small cell lung cancer**	N = 234	/	N = 200	/
Age (years), median (range)	60 (51–82)	/	60 (27–87)	0.11
Male sex, n (%)	157 (67.1)	/	126 (63.0)	0.37
Female sex, n (%)	77 (32.9)	/	74 (37.0)	0.37
Ethnicity (n)	Chinese (234)	/	Chinese (200)	/
Smoking history, n (%)	91 (38.9)	/	74 (37.0)	0.73
Drinking history, n (%)	77 (32.9)	/	71 (35.5)	0.50
Clinical stage (Early, Advanced)	/	/	76, 107	/
**Gastric cancer**	N = 34	/	N = 29	/
Age (years), median (range)	40 (28–54)	/	55 (41–67)	<0.05
Male sex, n (%)	18 (52.9)	/	20 (69.0)	0.20
Female sex, n (%)	16 (47.1)	/	9 (31.0)	0.20
Ethnicity (n)	Chinese (34)	/	Chinese (29)	/
Clinical stage (Early, Advanced)	/	/	15, 14	/
**Esophageal cancer**	N = 34	/	N = 61	/
Age (years), median (range)	40 (28–54)	/	60 (46–73)	<0.05
Male sex, n (%)	18 (52.9)	/	53 (86.9)	<0.05
Female sex, n (%)	16 (47.1)	/	8 (13.1)	<0.05
Ethnicity (n)	Chinese (34)	/	Chinese (61)	/
Smoking history, n (%)	/	/	42 (68.9)	/
Drinking history, n (%)	/	/	44 (72.1)	/
Clinical stage (Early, Advanced)	/	/	21, 27	/

**Validation cohort**				

**Ovarian cancer**	N = 20	N = 20	N = 20	/
Age (years), median (range)	54 (26–76)	51 (34–60)	52.5 (20–79)	0.57
Female sex, n (%)	20 (100.0)	20 (100.0)	20 (100.0)	/
Ethnicity (n)	Chinese (20)	Chinese (20)	Chinese (20)	/
Pausimenia, n (%)	/	11 (55.0)	14 (70.0)	0.34
Clinical stage (Early, Advanced)	/	/	8, 12	/
**Non-small cell lung cancer**	N = 75	/	N = 75	/
Age (years), median (range)	55 (38–77)	/	60 (36–80)	<0.05
Male sex, n (%)	54 (72.0)	/	44 (58.7)	0.09
Female sex, n (%)	21 (28.0)	/	31 (41.3)	0.09
Ethnicity (n)	Chinese (75)	/	Chinese (75)	/
Smoking history, n (%)	27 (36.0)	/	29 (38.7)	0.74
Drinking history, n (%)	27 (36.0)	/	26 (34.7)	0.96
Clinical stage (Early, Advanced)	/	/	38, 33	/
**Gastric cancer**	N = 29	/	N = 23	/
Age (years), median (range)	58 (46–69)	/	57 (43–66)	0.54
Male sex, n (%)	16 (55.2)	/	14 (60.9)	0.69
Female sex, n (%)	13 (44.8)	/	9 (39.1)	0.69
Ethnicity (n)	Chinese (29)	/	Chinese (23)	/
Clinical stage (Early, Advanced)	/	/	12, 11	/
**Esophageal cancer**	N = 29	/	N = 61	/
Age (years), median (range)	58 (46–69)	/	63 (48–82)	<0.05
Male sex, n (%)	16 (55.2)	/	56 (91.8)	<0.05
Female sex, n (%)	13 (44.8)	/	5 (8.2)	<0.05
Ethnicity (n)	Chinese (29)	/	Chinese (61)	/
Smoking history, n (%)	/	/	43 (70.5)	/
Drinking history, n (%)	/	/	39 (63.9)	/
Clinical stage (Early, Advanced)	/	/	17, 24	/

The p values were calculated using Analysis of variance (for the OC group) or two-sided Wilcoxon rank-sum test (for NSCLC, GC, and EC groups).

### N-glycome abundance distribution in ovarian cancer group

**Figure 2:**
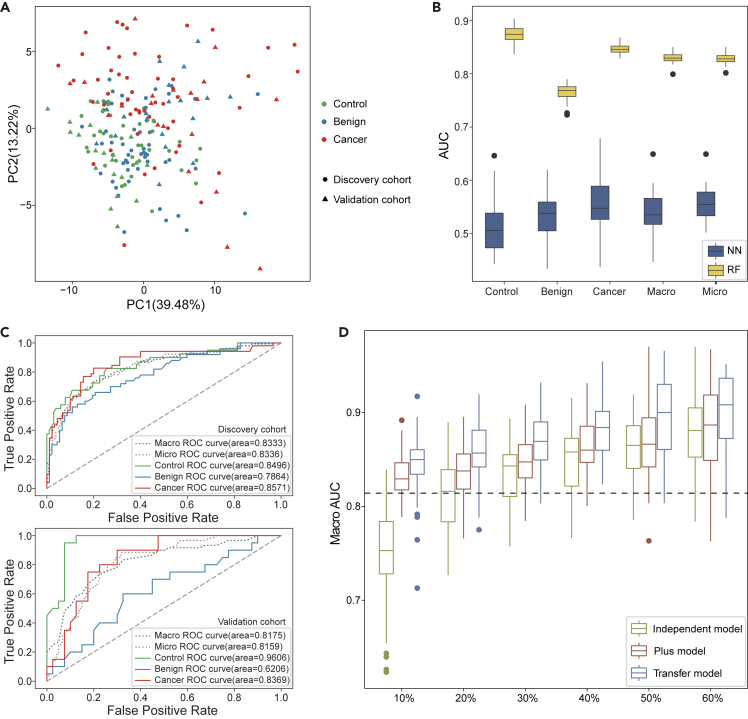
Transfer model achieved the best classification performance using N-glycome’ features in the validation cohort of ovarian cancer group (A) Principal component analysis of healthy control, benign neoplasm, and cancer patient samples in discovery and validation cohorts. (B) Comparison of classification performance on discovery cohort between neural networks and random forests. NN, neural networks; RF, random forests. (C) Area under the receiver operating characteristic curves show the classification performance of base model on the discovery cohort (up) and validation cohort (bottom). (D) Macro AUC of Independent model, Plus model and Transfer model applied on the test set of validation cohort. The x axis shows the proportion of the validation cohort using as the train/transfer set to generate the model. The dashed line shows the median Macro AUC of the base model applied on the test set of validation cohort.

Principal component analysis (PCA) was used to visualize the serum N-glycome’ features distribution of healthy control, benign neoplasm, and cancer patient samples in discovery and validation cohorts of ovarian cancer group (Figure 2A). As shown in the figure, there was a clear distinction between the cancer group and other groups, while the control group and benign group were mixed together and difficult to distinguish, indicating that patients with ovarian cancer had obvious changes in N-glycome patterns compared with benign patients. In addition, the PCA results revealed some differences between the discovery cohort and the validation cohort, such as the separation of benign groups, which frequently renders the results obtained from the discovery cohort are not completely applicable to the validation cohorts.

### Development of N-glycome-based multi-class diagnosis model

Based on the discovery cohort, we trained different machine learning multi-class classifiers (neural network (NN) and random forest (RF)) to classify the healthy control, benign neoplasm, and cancer patient samples using serum N-glycome’ abundance data from the training set (80% samples with the same class proportions as the cohort) and presented their final performance from the withheld test set (20% samples). 5-fold cross-validation method was used to evaluate their average classification performance. Compared to the NN model, whose AUROC scores were between 0.5 and 0.6, the RF multi-class model achieved a far better performance with AUROC scores around 0.8 (Figure 2B; Table S1). Therefore, the RF multi-class model was used as the base model for further analyses.

### Disparities in machine learning model performance

We then applied the base model to the validation cohort and compared its performance with that in the discovery cohort. The RF multi-class model showed a decent classification performance in the discovery cohort, with a mean Macro AUROC score of 0.83, and mean Micro AUROC score of 0.83 (Figure 2C), suggesting that patients with ovarian cancer detection based on serum N-glycome is feasible. In the validation cohort, however, the model’s diagnostic performance deteriorated, as shown by Macro AUROC and Micro AUROC scores less than 0.82 (Figure 2C; Table S2). This decline was particularly evident in distinguishing the benign neoplasm patients (one versus the others), with an AUROC score of only 0.62 in the validation cohort compared to 0.79 in the discovery cohort, which corresponded to the obvious separation between the benign group of the two cohorts in the previous PCA results. In addition, we noticed that the RF multi-class model achieved an AUROC score of 0.96 for healthy control samples in the validation cohort. This is possibly due to the high similarity between the control groups in the discovery and validation cohorts, and the small sample size of the validation cohort may also contribute to it.

### Transfer learning for improving model performance

To attain better diagnostic performance in the validation cohort, we tried the transfer learning scheme, which can provide improved performance by leveraging the knowledge learned from the discovery cohort. We randomly divided the validation cohort data of n% (n = 10, 20, 30, 40, 50, 60) as the transfer set and the remaining data as the test set, then performed assessments for three models: (1) Independent model: ab initio training and testing the RF multi-class model on the transfer set and the test set of the validation cohort, respectively. (2) Plus model: ab initio training the RF multi-class model using the Plus dataset including all the discovery cohort data and the validation cohort transfer set and testing it on the test set of the validation cohort. (3) Transfer model: ab initio training the RF multi-class model using discovery cohort data, followed by applying transfer learning to the transfer set from the validation cohort, and then testing it on the test set of the validation cohort. We found that the performance of the three models in the validation cohort was greatly improved over the base model even when only a small proportion of the validation cohort data was used as the transfer set, especially for the transfer models (Figures 2D and S1; Table S3). For example, with 10% of validation cohort data, transfer model could achieve a mean Macro AUROC of 0.84 and a mean Micro AUROC of 0.83 on the test set of validation cohort, which is comparable to the performance on the discovery cohort. This result suggests that the transfer model has successfully generalized its learned patterns to the validation cohort. It is an indication of transfer model’s ability to adapt to varying data distributions and implies its potential for practical applications in broader clinical settings. We then compared machine learning schemes on performance for the test set of validation cohort and found that transfer learning produced models with significantly better performance compared to the models from independent learning (e.g., P_Macro AUROC_ = 3.6e-07, P_Micro AUROC_ = 3.6e-08 for 30% partitions) and plus learning (e.g., P_Macro AUROC_ = 2.0e-03, P_Micro AUROC_ = 1.4e-02 for 30% partitions). Due to the absence of additional data from the discovery cohort, the performance of the independent learning is highly dependent on the sample size of the validation cohort. When trained with 10% of validation cohort data, the independent learning fails to produce reliable models, with a mean macro AUROC of only 0.75. As the dataset used for training increases, the independent model could achieve diagnostic performance similar to the plus model (e.g., P_Macro AUROC_ = 0.51, P_Micro AUROC_ = 0.18 for 50% partitions), but both are inferior to the transfer model. When using 60% of validation cohort data as the transfer set, the transfer model demonstrated excellent performance in the test set of the validation cohort, with Macro AUROC and Micro AUROC reaching approximately 0.90 and 0.89, respectively. Notably, transfer learning also significantly improved the classification performance of the benign group in the validation cohort compared to the base model (e.g., p = 8e-10 for 30% partitions, one versus the others), with the transfer model achieving a maximum mean AUC of 0.77 (Figure S2). In summary, transfer learning significantly improved the diagnostic performance of the base model on the validation cohort and achieved decent performance compared to the plus learning and independent learning schemes, even only with a small amount of validation data.

### Identification of a serum N-glycans biomarker panel for ovarian cancer

**Figure 3:**
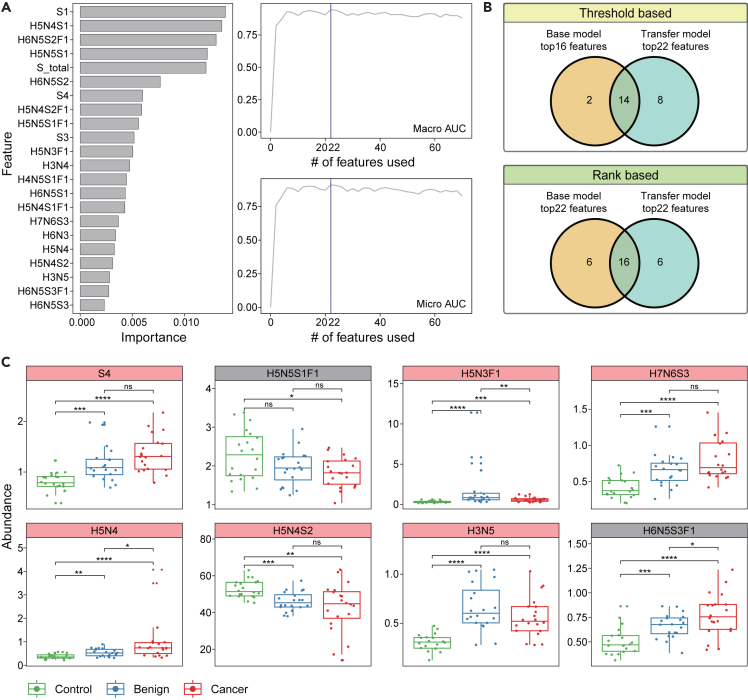
Transfer model captures potential N-glycome biomarkers specific to the validation cohort of ovarian cancer (A) Permutation importance illustrating the influence of individual N-glycome features on the classification performance of transfer model (left). Top22 features are shown. Variation of the evaluation metrics of the transfer model trained using different number of features (right). (B) Venn diagram showing the specific N-glycome features for the transfer model obtained by threshold and rank based criteria, respectively. Threshold based, the features that yielded the best model performance. Rank based, the features that ranked in the top 22. (C) 8 specific features abundance changes across healthy control, benign neoplasm, and cancer patient groups in validation cohort. The 6 features highlighted in pink were identified using the rank-based criteria. Statistical significance was determined using the p value of two-sided Wilcoxon rank-sum test. ns, p > 0.05; ∗, p<=0.05; ∗∗, p<=0.01; ∗∗∗, p<=0.001; ∗∗∗∗, p<=0.0001. H, hexose; N, *N*-acetylglucosamine; F, fucose; S, sialic acid.

Next, we selected the serum N-glycans and glycan derived traits, which contributing for the transfer model, based on the feature permutation importance to identity clues to model interpretability. Taking the model’s Macro AUROC and Micro AUROC into account, we finally determined the top 22 features including 18 serum N-glycans and 4 glycan derived traits to be the potential biomarkers (Figure 3A). These serum N-glycans and glycan derived traits achieved a mean Macro AUROC of 0.95 and a mean Micro AUROC of 0.91 in the validation cohort. As expected, significant changes in the abundance of them were observed between controls and patients with ovarian disease in the validation cohort (Figure S3). Interestingly, the 4 glycan derived traits were all sialylation, and two of them (tri-sialyation, tetra-sialylation) were significantly increased (p < 0.05) in patients with ovarian cancer, corresponding to previous research.^15^^,^^37^^,^^38^ However, the total sialylation was substantially changed in patients with ovarian cancer in the opposite trend, possibly due to the decrease abundance of some low sialylated N-glycans. For the N-glycans, it was found that 10 N-glycans, including H6N5S2F1, H5N3F1, H3N4, H4N5S1F1, H6N5S1, H7N6S3, H6N3, H5N4, H3N5, and H6N5S3F1, were significantly increased (p < 0.05) in patients with ovarian cancer compared with healthy controls. Moreover, three of them (H6N5S2F1, H5N4, and H6N5S3F1) were still significantly increased from benign neoplasm to patients with cancer, implicating their contribution to OC metastasis and the potential to be the basis for distinguishing patients with ovarian cancer from healthy controls and benign neoplasm. Notably, two N-glycans (H5N3F1, H3N4) showed a discontinuous pattern of abundance changes, with a significantly increased abundance from healthy controls to benign neoplasm group, and then a significant decrease in patients with ovarian cancer. In addition, we also found three N-glycans (H5N4S2F1, H5N4S1F1, H5N4S2) were significantly down-regulated in patients with ovarian disease, implicating their inhibitory role in ovarian cancer progression.

### Specific N-glycans biomarker for the validation cohort

Using the same criteria for selection, we obtained the top 16 features consist of 13 N-glycans and 3 glycan derived traits for the base model (Figure S4). Comparing the contributing features between the base model and transfer model, it was found that there were 8 features that were unique to the transfer model (Figure 3B). We further compared the top 22 features from the base model with transfer model and the result showed that there were still 6 features that only contributed to the transfer model (Figure 3B). Specifically, the abundance of the 6 features, including S4, H5N3F1, H7N6S3, H5N4, H5N4S2, and H3N5, differed significantly between healthy controls and patients with ovarian cancer in the validation cohort (Figure 3C), while no such significant difference was observed in the discovery cohort except for H5N3F1 (Figure S5). Overall, these results suggest that the transfer learning can capture N-glycans features that are specific to the validation cohort, which may explain the improved performance of the transfer model and is of great significance for the diagnosis of patients with ovarian cancer in the validation cohort.

### Validation of transfer scheme for other serum-glycan-based cancer prediction

**Figure 4:**
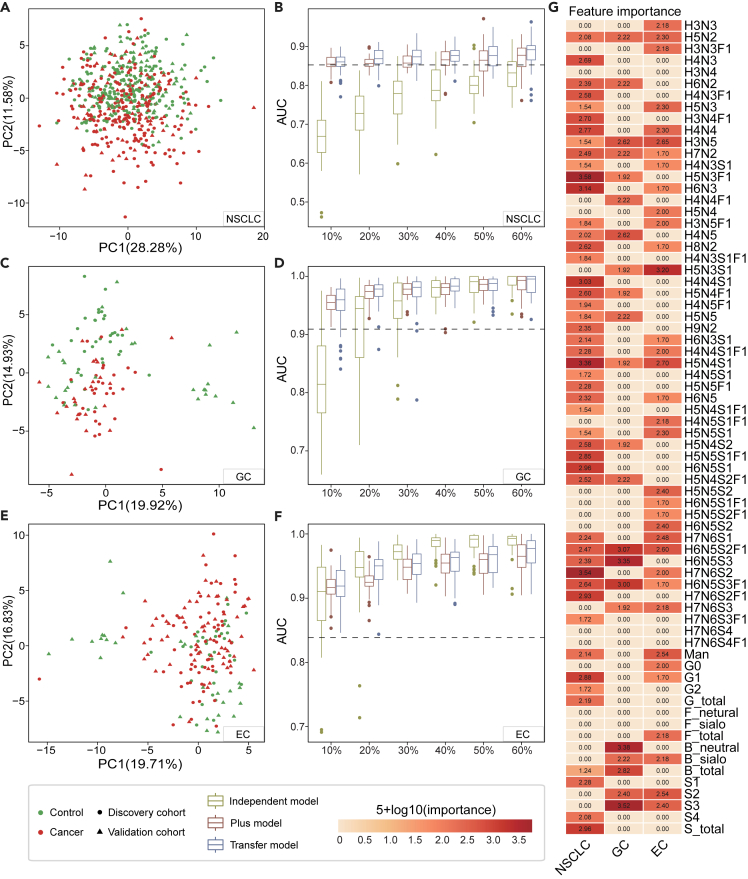
N-glycome features-based transfer learning for other cancer groups (A, C, and E) Principal component analysis of healthy control and cancer patient samples in discovery and validation cohorts of NSCLC group (A), GC group (C) and EC group (E). NSCLC, Non-small-cell lung cancer; GC, gastric cancer; EC, esophageal cancer. (B, D, and F) AUC score of Independent model, Plus model and Transfer model applied on the test set of validation cohort of NSCLC group (B), GC group (D) and EC group (F). The x axis shows the proportion of the validation cohort using as the transfer set to generate the model. The dashed line shows the median AUC score of the base model applied on the test set of validation cohort. (G) Heatmap shows the permutation importance of N-glycome features in different disease groups. The redder, the more important. The score displayed was calculated by the formula: 5+log10 (importance). Features with negative importance were set a score of 0. NA, the feature is not present in this disease group. H, hexose; N, N-acetylglucosamine; F, fucose; S, sialic acid.

Furthermore, we applied the same strategies on the serum samples from non-small cell lung (NSCLC), gastric (GC) and esophageal cancers (EC), which only include patients with cancer and healthy controls (Table 1). Principal component analysis results indicated obvious differences of validation cohorts from the discovery cohorts (Figures 4A, 4C, and 4E), corresponding to the decrease of performance of the base model on the validation cohort (Figure S6; Table S2). Then, using transfer learning scheme, we successfully improved the classification performance for all three cancer groups with a higher mean AUC score (Figures 4B, 4D, and 4F; Tables S4, S5, and S6). Compared to independent model and plus model, transfer model exhibited the best performance in NSCLC groups, with the AUC score around 0.90 (Figure 4B). For GC and EC groups, transfer learning showed better performance than independent learning and plus learning when the transfer set was small, demonstrating the superiority of transfer learning in the data-disadvantaged situation. As the dataset size increased, the independent model got improved AUC score, approached or even exceeded that of the transfer model (Figures 4D and 4F). In the EC group, for example, with 30% or more of the validation cohort data for training, independent model achieved a mean AUC score of 0.9717, significantly higher than the transfer model’s AUC score (p = 3.3e-05). These results indicated that the ultimate solution to improve the model performance would be to increase the number of samples for model training. Notably, the control and cancer samples are not balanced in the validation cohort of EC group, which may influence the evaluation of the model performance. We resampled the cancer samples to form a data-balanced cohort to reevaluate the model performance and the result supported above conclusions (Table S7). Moreover, we identified specific N-glycans and glycan derived traits that can classify different cancers based on the feature importance for transfer model (Figure 4G). The abundance of four N-glycans, H5N3F1, H7N6S2, H5N4S1, and H6N3, made great contributions to the classification of healthy controls and patients with NSCLC. For the subjects in the GC group, S3, B_neutral, and H6N5S3 showed higher importance, while H5N3F1 was found to significantly influence the model’s diagnostic performance for patients with EC. In summary, for the binary classification tasks of NSCLC, GC, and EC diseases, the transfer model can also achieve improved performance on the validation cohort and show superiority than independent and plus learning scheme in the data-disadvantaged situation. With transfer model, we can identify disease-specific N-glycans signatures, which are expected to become diagnostic biomarkers for the patients with cancer.

## Discussion

Previous studies have reported that the N-glycome-based classification models can accurately distinguish between healthy individuals and patients with cancer,^39^^,^^40^ demonstrating reliable predictive performance. However, the heterogeneity of data between different cohorts can lead to a decrease in model performance when predicting across cohorts. We observed the AUC values of the base model trained on the discovery cohort decreased by 0.02–0.1 in the validation cohort (Figures 2C and S6), which affects the generalizability and applicability of the model.

In this work, we showed the superiority of the transfer learning scheme in glycome based assisted cancer diagnosis. We found that transfer learning can provide a possible solution to enhance the performance of the model in new cohorts by transferring the shared knowledge learned from the training cohort. For example, it was found that the AUC score of the transfer model is around 0.05 to 0.1 higher than that of the base model in the ovarian cancer group (Figure 2D). Our results on various cancer groups had also supported that the transfer model achieved the highest AUC score in the validation cohort compared to the independent model and plus model. In addition, transfer learning demonstrated advantages in selecting glycan biomarkers associated with relevant cancers. We noticed that the transfer model was able to identify glycome biomarkers that are specific to the study cohort, which holds significant implications for advancing precision medicine.

**Figure 5:**
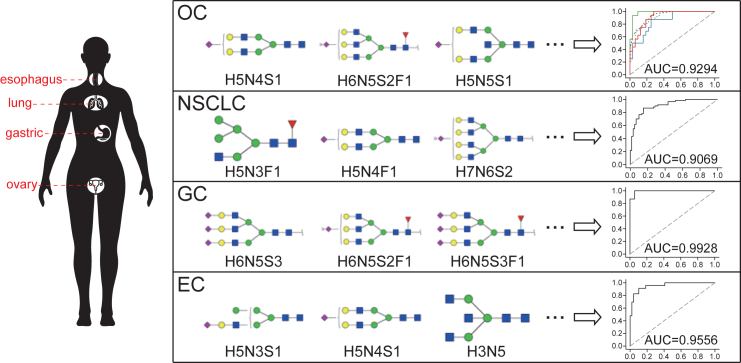
N-glycome features associated with different cancers and their potential for cancer diagnostic The N-glycome features that are contributed to the transfer model for different cancers. (top3 glycans were shown due to the space). Area under the receiver operating characteristic curves show the classification performance of the top10 N-glycome features on the validation cohort. For ovarian cancer, only the Macro AUC was shown in the figure, and the value of other AUC scores were: Control AUC = 0.9824, Benign AUC = 0.8535, Cancer AUC = 0.9121 and Micro AUC = 0.9145. H, hexose; N, N-acetylglucosamine; F, fucose; S, sialic acid.

Through the transfer model, we have identified the glycome features that are closely associated with different cancers (Figure 5). Sialoglycans, especially the tri-antennary and tetra-antennary sialylated N-glycans were significantly increased in the patients with OC. Several directly measured glycans containing sialic acid, such as H6N5S2F1, H7N6S3, and H6N5S3F1 also demonstrated an increased trend in patients with ovarian cancer compared with healthy controls. It has been reported that hypersialylation could enhance immune evasion and tumor cell survival, and stimulate tumor invasion and migration,^38^^,^^41^ which may contribute to the development and progression of ovarian cancer. Additionally, we have also observed a significant increase in glycans H3N4 and H3N5, as well as the derived trait G0 (measuring all agalactosylated glycans), corresponding to previous research.^42^ Malhotra et al. demonstrated that these particular agalactosyl G0 glycoform of IgG may elicit a pro-inflammatory response as a result of their ability to act as ligands for mannose binding lectin and subsequent complement activation.^43^^,^^44^^,^^45^ In another study by Bones et al., it was described that increased the expression of IgG carrying core fucosylated G0 type glycans with specificity against an antigen on the tumor cell surface may result in subsequent complement activation with tumor cell lysis via the membrane attack complex.^46^ It is noteworthy that, similar change in agalactosylated glycans was also reported implicated in other cancer progression, such as prostate^47^ and stomach^46^ cancer, indicating the great potential of the glycan derived trait G0 in cancer diagnosis and treatment. For NSCLC group, glycans including H5N3F1, H7N6S2, H5N4S1, and H6N3 demonstrated high feature importance in classifying the healthy controls and patients. Subjects with GC mainly have close connections with sialylated glycans and bisecting GlcNAcylated N-glycans, such as H6N5S3, H6N5S2F1, H6N5S3F1, H4N5, H3N5, and H4N5F1. A significant increase in bisecting GlcNAcylated N-glycans were found in the patients with gastric cancer (p = 8.2e-8, not shown). The bisecting GlcNAc plays an important role in retarding tumor progression through reducing galectin-lattice dependent growth factor signaling,^48^ indicating that it may be a part of disease pathophysiology. In EC group, H5N3S1 glycan was the most potential biomarker with far greater importance than other glycan features.

Using the top10 glycome features, transfer models demonstrated outstanding performance in the validation cohort of all four types of cancers. For all the four cancer groups, transfer models achieved an AUC score exceeding 0.90, which outperformed the traditional approach of PLS-DA (Figures 5 and S7; Table S8). These results highlighted the immense potential of transfer learning method in the diagnosis of patients with cancer and identification of promising cancer biomarkers. Overall, our study indicated that the introduction of transfer learning contributes to enhancing the clinical applications of glycome-based cancer diagnostic model, which could serve as a powerful approach for cancer risk assessment and identification of potential biomarkers, providing new perspectives for the diagnosis and treatment of various cancers. Given the poor performance of other learning schemes (base model, independent model and plus model) in the validation cohort (serving as data-disadvantaged cohort), our efforts could largely diminish the negative impacts on health care for the data-disadvantaged groups, while bridge the gap of health care disparities.

Previous studies have reported that glycosylation is strongly influenced by factors such as age and sex.^49^^,^^50^^,^^51^ To determine their specific influence on our findings, we resampled the validation cohort of NSCLC group and formed the age (age between 55 and 60) and sex (male only) subsets to re-evaluate the model performance. As the results shown, we found that there is indeed a slight difference in the model performance, but the conclusion that the transfer learning is superior to other schemes is still upheld (Tables S9 and S10). Another issue to be considered is the potential collinearity problems caused by the mixed features of single glycans and derived traits in the same model. We re-trained the independent, plus and transfer models using only the directly detected glycans and evaluated their performance in the validation cohort of the ovarian cancer group (Table S11). The results demonstrated a similar model performance and transfer model still got the highest AUC score than the independent and plus model, supporting the conclusion that transfer learning could improve diagnosis performance.

Despite of these limitations, this work represents the first of its kind to introduce transfer learning scheme in the building of glycome-based cancer diagnostic model, and have demonstrated its superiority in improving diagnosis performance and identifying cancer biomarkers. It has provided a novel reliable and universal perspective for future cancer diagnosis in clinics.

### Limitations of the study

As a pilot study, our work has several limitations. Given the low incidence of ovarian cancer in the general population,^52^ procurement of qualified specimens for modeling and biomarker discovery is challenging. While we acknowledge the limited sample size of subjects, we emphasize rigor in our statistical approach, adhering to the PCS framework^53^ for modeling and evaluation of model stability and robustness, as well as the use of independent validation cohort and repeated procedures. In our future work, we might attempt to include more samples to complement our results. Secondly, the differences across cohorts in this study mainly stem from variations in sampling time, and inclusion of study cohorts from different sampling locations may further highlight the advantages of transfer learning. Though we could not validate it at this moment, this hypothesis should be tested in the future. Also, there is limited biological evidence available to substantiate the identified associations between glycome and cancer, and future work to uncover the mechanisms underlying these associations is required to enhance our comprehension of the precise role played by cancer-specific glycans in the pathogenesis of cancer.

## STAR★Methods

### Key resources table

**Table:** 

REAGENT or RESOURCE	SOURCE	IDENTIFIER
**Biological samples**		

Serum from diagnosed OC, NSCLC, GC and EC patients, as well as corresponding healthy controls and benign patients	Tongji Hospital affiliated to Tongji Medical College of Huazhong University of Science & Technology	N/A

**Deposited data**		

Raw MS data	This paper	ProteomeXchange Consortium via the PRIDE (https://www.ebi.ac.uk/pride/) Dataset identifier: PXD040959; PXD040883

**Software and algorithms**		

Python (version 3.7.15)	Python Software Foundation	https://www.python.org/
Pandas (version 1.2.4)	Python package	RRID: SCR_018214; https://pandas.pydata.org/
PyTorch (version 1.11.0)	Python package	RRID: SCR_018536; https://pytorch.org/
scikit-learn (version 1.0.2)	Python package	RRID: SCR_002577; http://scikit-learn.org/
R (version 4.2.1)	R software	http://www.R-project.org
transfer-learning methods for random forests	Segev et al.^54^	https://github.com/Luke3D/TransferRandomForest
GlycoWorkBench (version 2.1)	GlycoWorkBench software	RRID: SCR_000782; https://code.google.com/p/glycoworkbench/

### Resource availability

#### Lead contact

Further information and requests for resources should be directed to and will be fulfilled by the lead contact, Kang Ning (e-mail: ningkang@hust.edu.cn).

#### Materials availability

This study did not generate new unique reagents.

#### Data and code availability

• All the raw data enrolled in this study have been deposited to the ProteomeXchange Consortium and are publicly available as of the date of publication. The accession number is listed in the key resources table.
• This paper does not report original code.
• Any additional information required to reanalyze the data reported in this paper is available from the lead contact upon request.

### Experimental model and study participant details

#### Study population

This study was performed in accordance with the principles of the Declaration of Helsinki criteria, and was approved by the Ethics Committee of Tongji Hospital affiliated to Tongji Medical College of Huazhong University of Science & Technology (study approval number: TJ-IRB20221109). All participants were recruited from the Tongji Hospital affiliated to Tongji Medical College of Huazhong University of Science & Technology. The study is exempt from informed consent, which has been approved by the Ethics Committee. There were four cancer groups, including ovarian cancer (OC) group, non-small cell lung cancer (NSCLC) group, gastric cancer (GC) group and esophageal cancer (EC) group. Cancer patients in OC group were enrolled with the following criteria: (i) age of ≥18 years, (ii) pathologically confirmed OC, and (iii) no history of other malignancies. Benign samples of OC group included patients with ovarian serous cystadenoma, mucinous cystadenoma and ovarian fibroma and followed the criteria: (i) age of ≥18 years, (ii) no history of other malignancies. Asymptomatic adults with non-OC were included as controls during the same recruitment period and the inclusion criteria were: (i) age of ≥18 years, and (ii) no history of malignancies. Subjects with NSCLC (or GC, EC) were enrolled with the following criteria: (i) age of ≥18 years, (ii) pathologically confirmed NSCLC (or GC, EC), and (iii) no history of other malignancies. The healthy controls of NSCLC (or GC, EC) group were recruited during the same recruitment period with the inclusion criteria: (i) age of ≥18 years, and (ii) no history of malignancies. Patients with severe cardiovascular diseases such as coronary heart disease and stroke were excluded. Each of the four cancer groups were divided into two cohorts according to the sample collection time: the discovery cohort and the validation cohort. The ovarian cancer group consisted of healthy controls (N_discovery_ = 40, N_validation_ = 20), benign neoplasm (N_discovery_ = 50, N_validation_ = 20), and cancer patients (N_discovery_ = 52, N_validation_ = 20). For non-small cell lung cancer (NSCLC), gastric cancer (GC) and esophageal cancer (EC) groups, there were only two classes: healthy controls and cancer patients. The NSCLC group consisted of 309 healthy controls (N_discovery_ = 234, N_validation_ = 75) and 275 cancer patients (N_discovery_ = 200, N_validation_ = 75). The GC group consisted of 63 healthy controls (N_discovery_ = 34, N_validation_ = 29) and 52 cancer patients (N_discovery_ = 29, N_validation_ = 23). And the EC group consisted of 63 healthy controls (N_discovery_ = 34, N_validation_ = 29) and 122 cancer patients (N_discovery_ = 61, N_validation_ = 61). The demographic and clinical characteristics of the participants were shown in Table 1. Each group was divided into two cohorts according to the sample collection time: the discovery cohort and the validation cohort. There was a smaller number of samples in the validation cohort, which served as the data-disadvantaged cohort.

### Method details

#### N-glycome data acquisition

N-glycome profile was characterized by a MALDI-MS based high-throughput analytical assay.^55^ Profiling of Serum N-glycans were performed with the same manner as reported in our recent study,^56^^,^^57^ including N-glycan release, chemical derivatization, MALDI-MS analysis and MS data processing.

In detail, human serum N-glycans were enzymatically released by PNGaseF digestion using Protein Deglycosylation Mix Ⅱ (New England Biolabs, Ipswich, MA, USA), and subsequently separated and purified through solid-phase extraction (SPE).^20^ First, serum samples (10 μL) were dissolved in ultra-purified water, followed by the addition of 3.6 μL of PNGaseF buffer and 2.4 μL of denaturing buffer to obtain a reaction mixture of 100 μL. The mixture was denatured in a Thermo-Shaker (Ningbo Hinotek Technology Co., Ltd) and then cooled to room temperature before 12 μL of NP-40 and 5 units of PNGaseF were sequentially introduced. The resulting mixture was incubated at 37°C overnight. The glycans were then captured by solid-phase extraction (SPE) with porous graphitized carbon (PGC, Sigma-Aldrich, St. Louis, MO, USA), and further concentrated using a vacuum concentrator (Eppendorf, Germany). To prevent the loss of sialic acid during MALDI-MS analysis, we performed methylamidation of the carboxyl group in the glycans according to our previous study.^58^ In brief, we dissolved the sample in 25 μL of dimethyl sulfoxide (DMSO) containing 1 M methylamine hydrochloride and 0.5 M N-methylmorpholine. Next, we added 25 μL of DMSO containing 50 mM (7-azabenzotriazol-1-yloxy) trispyrrolidinophosphonium hexa-fluorophosphate (PyAOP). After incubating the mixture at room temperature for 30 min, the derivatized glycans were purified by solid-phase extraction (SPE) using microcrystalline cellulose (MCC, Sigma-Aldrich, St. Louis, MO, USA).^59^

For MALDI-MS detection, the 5800 MALDI-TOF-MS (AB Sciex, Concord, Canada) was used to analyze the glycome spectrum. In brief, the purified sample was solubilized in 5 μL of 50% ACN aqueous solution, and then 0.5 μL MALDI sample was added to the MALDI plate. After air-drying, an equal volume of 10 mg/mL 2,5-Dihydroxybenzoic acid (DHB, Sigma-Aldrich, St. Louis, MO, USA) containing 50 mM sodium acetate (Sigma-Aldrich, St. Louis, MO, USA) was added. The range of m/z was monitored at 1000–4500, and a total of 1000 laser shots were applied to each sample spot. MS data were acquired in the positive ion reflector mode. The samples were randomly spotted onto the plate in triplicate to mitigate the batch effect. Glycan structures were assigned referring to GlycoMod and previous literatures.^60^^,^^61^^,^^62^ Moreover, nanoLC-PGC-MS/MS analysis was employed to validate the N-glycans structures.^58^^,^^63^ GlycoWorkBench 2.1 software was used for the visualization of glycan structures.

All the raw data enrolled in this study have been deposited to the ProteomeXchange Consortium. Serum N-glycome profile and glycan derived traits have been illustrated in the supplemental information (Tables S12 and S13).

#### Machine learning for diseases diagnosis

Machine learning models are implemented by Python 3.7.15 using standard libraries that are publicly available: pandas (1.2.4), PyTorch (1.11.0) and scikit-learn (1.0.2). For each cancer group, samples in discovery cohort were randomly divided into a training set (80% of samples) and a test set for independent evaluation (remaining 20%). Random forests (RF) and neural network (NN) were trained as classifier models for the diagnosis of cancer patients by using serum N-glycome profiles. We implemented the RF classifier with the following parameter: ntree = 500. For NN classifier, there were 4 layers: an input layer, a fully connected layer with 200 nodes, a fully connected layer with 100 nodes, and an output layer. A cross-validation procedure was applied to determine the within-training set performance by splitting data into training and test sets for 40-times repeated, fivefold-stratified cross-validation. The optimal models selected based on cross-validated results were then evaluated in the validation cohort dataset and AUROC value was calculated accordingly for the visualization of results.

#### Transfer learning

For transfer learning, we set the discovery cohort as the source context and the validation cohort as the target context. We randomly divided n% (n = 10, 20, 30, 40, 50, 60) of validation cohort samples as transfer set and the remaining samples as the test set. The base model was *ab initio* trained in the source context with the function *RandomForestClassifier* integrated in the scikit-learn package with default parameters, followed by applying transfer learning to the transfer set from the target context. We applied two random forest transfer algorithms to each decision tree in the base model: (1) The Structure Expansion Reduction (SER) algorithm searches greedily for locally optimal modifications of each tree structure by trying to locally expand or reduce the tree around individual nodes.^54^ For each node *v* in the decision tree of base model, a set of all labeled points in the target data that reaches *v* was calculated. Then, the leaf *v* is expanded to a full tree with respect to the calculated sample set, and the internal node *v* will be decided whether to prune into a leaf node under the consideration of classification error. (2) The Structure Transfer (STRUT) algorithm does not modify structure, but only the parameter (thresholds) associated with decision nodes.^54^ It adapts a decision tree trained on the source context to the target context by discarding all numeric threshold values in the tree and re-trained a new threshold for a node *v* using the subset of target samples that reach *v*. The two types of modified decision trees were then mixed together to obtain the final transfer random forest, which is defined as the transfer model and used for prediction. Cross-validation was repeated 40-times to obtain a distribution of transfer random forest prediction evaluations on the test set of validation cohort, representing the transfer models’ diagnosis performance.

#### Potential biomarker identification

To identify the N-glycans and glycan derived traits that contributed the most to diagnosis, two major aspects were considered for the model. Firstly, we ranked the glycans features according to the permutation importance, which is defined to be the decrease in a model score when a single feature value is randomly shuffled.^64^ The calculation of permutation importance was repeated 40-times and the average value was determined as the final importance score. Next, we gradually selected the topN features according to the permutation importance to re-build the model and recorded the model performance for 40-times repeats. The glycans features used to achieve the best average performance were then identified to be the potential biomarkers.

### Quantification and statistical analysis

#### Machine learning performance evaluation

We included Macro AUROC and Micro AUROC to characterize the model performance in ovarian cancer group as our model provided outputs of probabilities for three disease phenotypes (control, benign, cancer), which formed a tri-classification task. Macro AUROC evaluated the average classification performance of three disease phenotypes, while Micro AUROC focused on whether each sample was classified accurately. In addition, we also considered AUROC for each of the three disease phenotypes (one versus all others) to understand the model’s ability in discriminating a particular class from others. For NSCLC, GC and EC groups, the model performance was evaluated using AUROC value, which considers the trade-offs between sensitivity and specificity at all possible thresholds. Another widely used machine learning performance metric is the AUPR value, which has been mathematically proven that the performance ranks of two models remain same in the ROC space and the PR space. However, linear interpolation in the precision–recall space is problematic, which may lead to inaccurate calculation of AUPR for datasets of small sample sizes. Thus, AUROC is a more robust metric for evaluating machine learning performance in our study.^65^ Also, we have included other evaluation metrics including precision, recall, f1-score and accuracy as a reference for performance evaluation.

#### PCS framework

Throughout the statistical analysis, we adhered to the PCS (Predictability, Computability and Stability) framework for trustworthy data science, which has been used and proven valuable in many previous scientific discoveries including interpretable drug response prediction^66^ and metabolomic signature of pancreatic cancer risk.^67^ For the modeling stage as in this work, the PCS (Predictability, Computability and Stability) framework uses predictability as a reality check, and for reproducibility, it advocates for a stability analysis across different reasonable perturbations of the data and models that pass the prediction check. Under this framework, the entire discovery cohort was divided into training set and test set for base model training and selecting. The set-aside validation cohort was used for obtaining an unbiased evaluation of the three learning schemes. Moreover, a 40-times repeats procedures were applied for all model evaluation processes to guarantee the stability of the results.

## Supplemental information

Document S1. Figures S1–S7 and Table S13

Table S1. Performance index values of neural networks (NN) and random forests (RF) on the discovery cohort of ovarian cancer group, related to Figure 2

Table S2. Performance index values for the base model on the discovery cohort and validation cohort, related to Figure 2

Table S3. Performance index values of Independent model, Plus model and Transfer model applied on the test set of validation cohort of ovarian cancer group, related to Figure 2

Table S4. Performance index values of Independent model, Plus model and Transfer model applied on the test set of validation cohort of non-small cell lung cancer group, related to Figure 4

Table S5. Performance index values of Independent model, Plus model and Transfer model applied on the test set of validation cohort of gastric cancer group, related to Figure 4

Table S6. Performance index values of Independent model, Plus model and Transfer model applied on the test set of validation cohort of esophageal cancer group, related to Figure 4

Table S7. Performance index values of Independent model, Plus model and Transfer model applied on a balanced test set of validation cohort of esophageal cancer group, related to Figure 4

Table S8. Performance index values of the Transfer model using top10 N-glycome features on the test set of validation cohort, related to Figure 5

Table S9. Performance index values of Independent model, Plus model and Transfer model applied on a sub-test set (male only) of validation cohort of non-small cell lung cancer group, related to Figure 4

Table S10. Performance index values of Independent model, Plus model and Transfer model applied on a sub-test set (age between 55 and 60) of validation cohort of non-small cell lung cancer group, related to Figure 4

Table S11. Performance index values of Independent model, Plus model and Transfer model applied on the test set of validation cohort of ovarian cancer group (using features of single glycans), related to Figure 2

Table S12. MALDI-MS spectra of N-glycans identified in human serum, related to STAR Methods
