# Supplementary material for: Deep learning enhanced the diagnostic merit of serum glycome for multiple cancers
Source: iScience. 2023 Dec 13;27(1):108715. doi: 10.1016/j.isci.2023.108715 (PMC10788220; doi:10.1016/j.isci.2023.108715)
Supplement: Document S1. Figures S1–S7 and Table S13 [file mmc1.pdf]

iScience, Volume *27*

Supplemental information

Deep learning enhanced the diagnostic

merit of serum glycome for multiple cancers

Haobo Zhang, Si Liu, Yi Wang, Hanhui Huang, Lukang Sun, Youyuan Yuan, Liming Cheng, Xin Liu, and Kang Ning

Supplemental Information

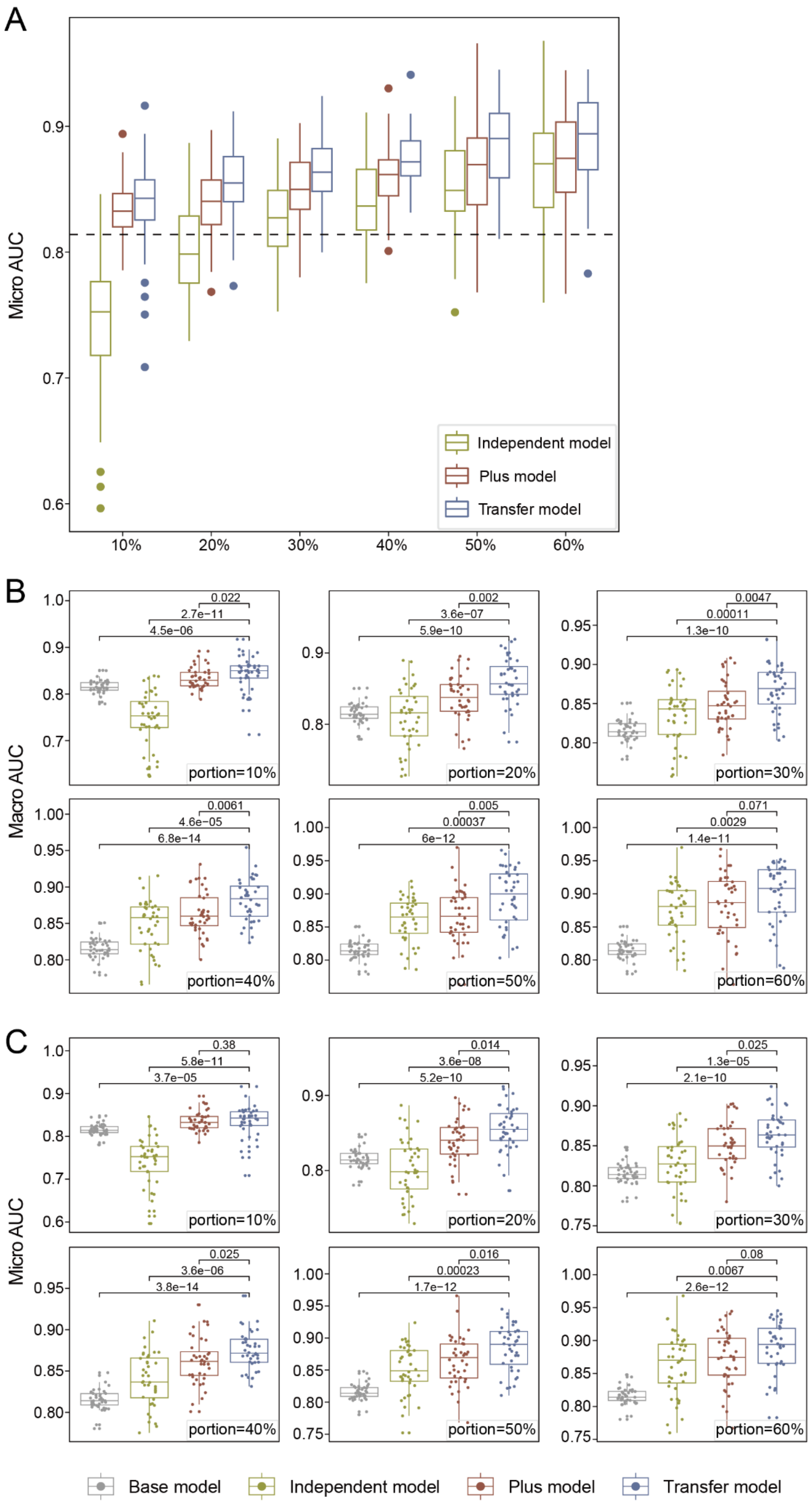

Figure S1. Comparison of different models and split ratio for healthy control, benign neoplasm, and cancer patients classification using N-glycome features in validation cohort of ovarian cancer group, related to Figure 2. (A) Micro AUC of Independent model, Plus model and Transfer model applied on the test set of validation cohort. The x-axis shows the proportion of the validation cohort using as the transfer set to generate the model. The dashed line shows the median Micro AUC of the base model applied on the test set of validation cohort. **(B, C,)** Macro AUC (B) and Micro AUC (C) of Base model, Independent model, Plus model and Transfer model trained using different proportion of validation cohort dataset. The p values were calculated using two-sided Wilcoxon rank sum test.

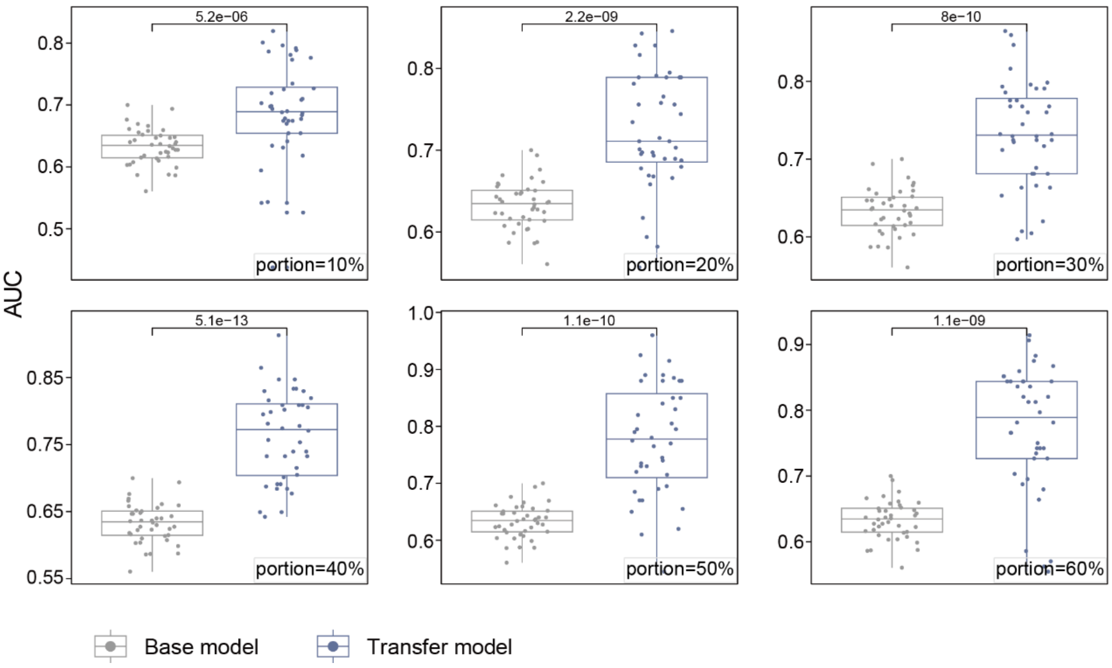

Figure S2. Comparisons of AUC score obtained by base model and transfer model to distinguish benign neoplasm and other subjects using N-glycome features in validation cohort of ovarian cancer group, related to Figure 2. The p values were calculated using two-sided Wilcoxon rank sum test.

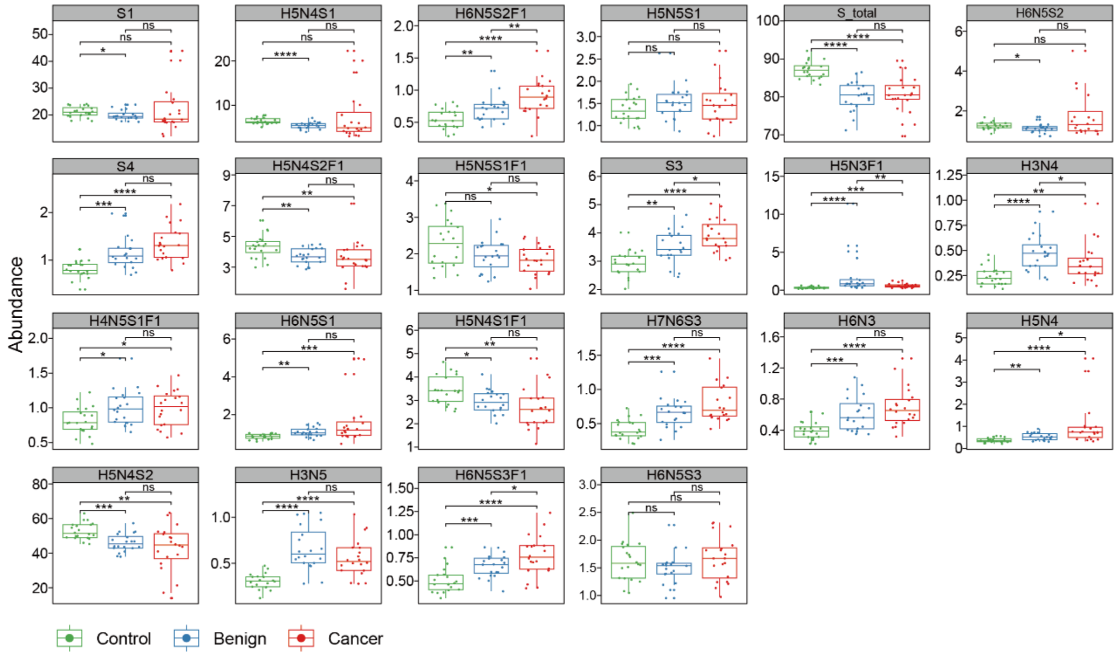

Figure S3. Abundance changes of the top22 N-glycans feature for transfer model across healthy control, benign neoplasm and cancer patient groups in validation cohort, related to Figure 3. Statistical significance was determined using the p-value of two-sided Wilcoxon rank sum test. ns, p>0.05; *, p<=0.05; **, p<=0.01; ***, p<=0.001; ****, p<=0.0001. H, hexose; N, N-acetylglucosamine; F, fucose; S, sialic acid.

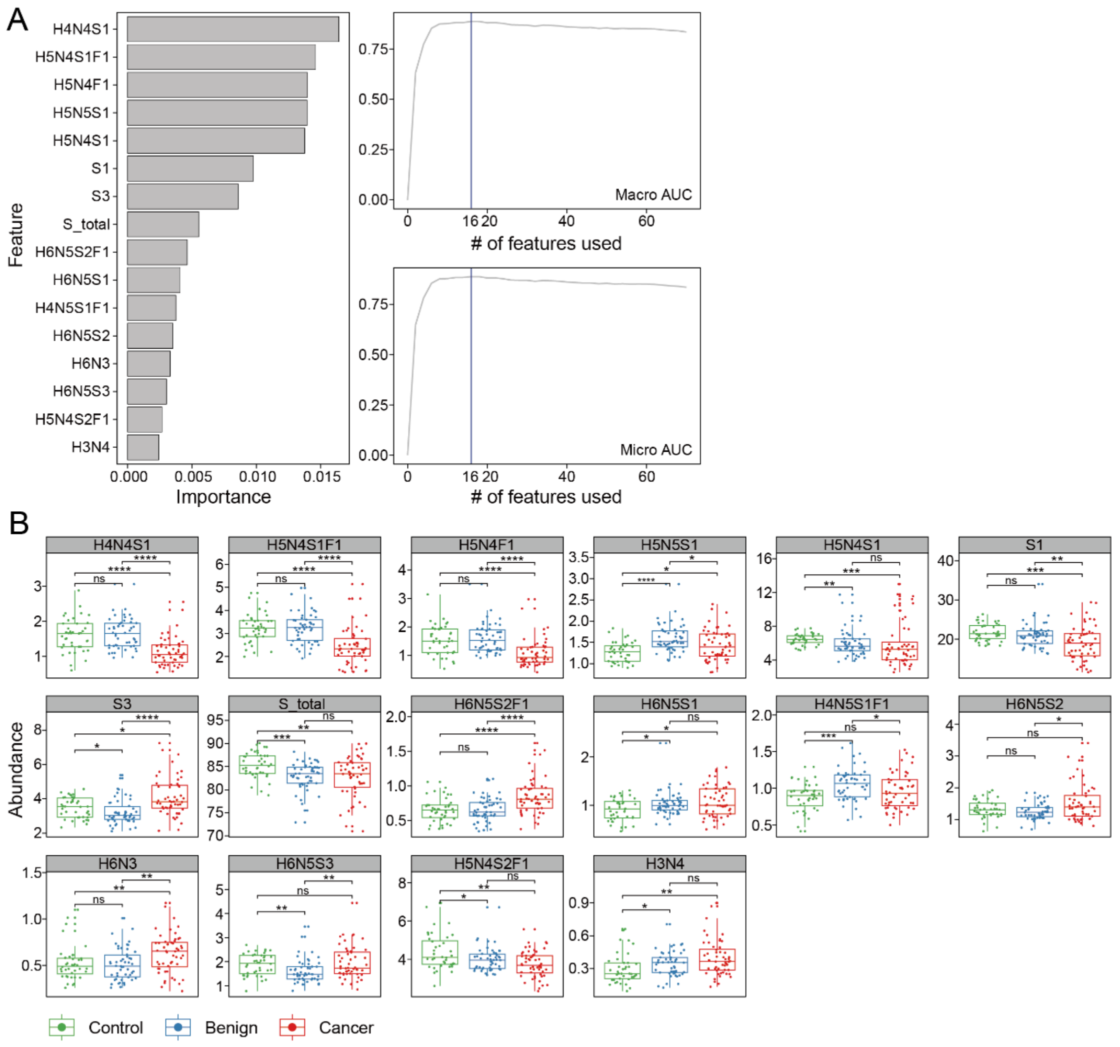

Figure S4. Potential N-glycome biomarkers of ovarian cancer identified by base model, related to Figure 3. (A) Permutation importance illustrating the influence of individual N-glycome features on classification performance of base model (left). Top16 features are shown. Variation of the evaluation metrics of the base model trained using different number of features (right). **(B)** Abundance changes of top16 N-glycome features for base model across healthy control, benign neoplasm, and cancer patient groups in discovery cohort. Statistical significance was determined using the p-value of two-sided Wilcoxon rank sum test. ns, p>0.05; *, p<=0.05; **, p<=0.01; ***, p<=0.001; ****, p<=0.0001. H, hexose; N, N-acetylglucosamine; F, fucose; S, sialic acid.

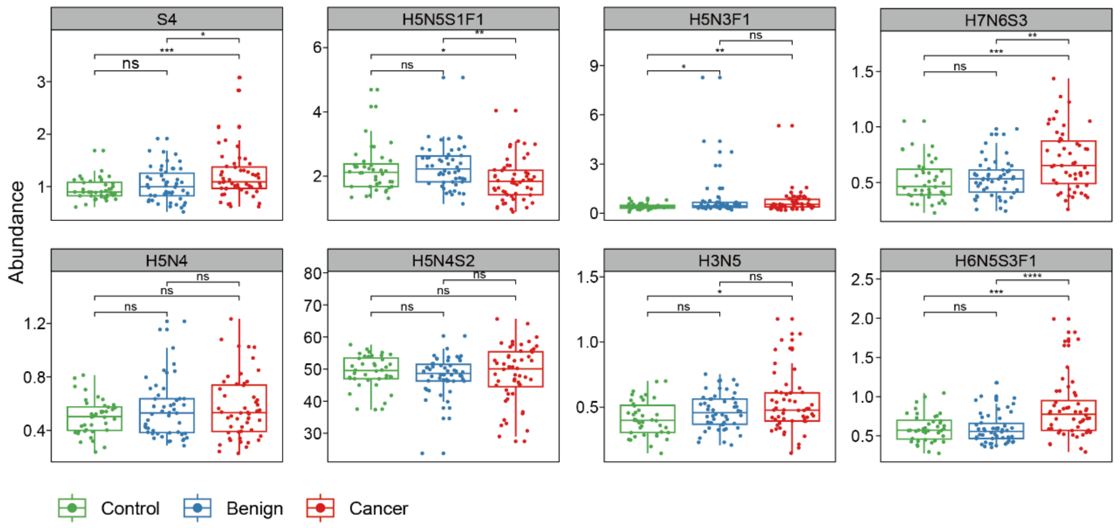

Figure S5. Abundance changes of the 8 specific N- glycome features for transfer model across healthy control, benign neoplasm, and cancer patient groups in discovery cohort, related to Figure 3. Statistical significance was determined using the p-value of two-sided Wilcoxon rank sum test. ns, p>0.05; *, p<=0.05; **, p<=0.01; ***, p<=0.001; ****, p<=0.0001. H, hexose; N, N-acetylglucosamine; F, fucose; S, sialic acid.

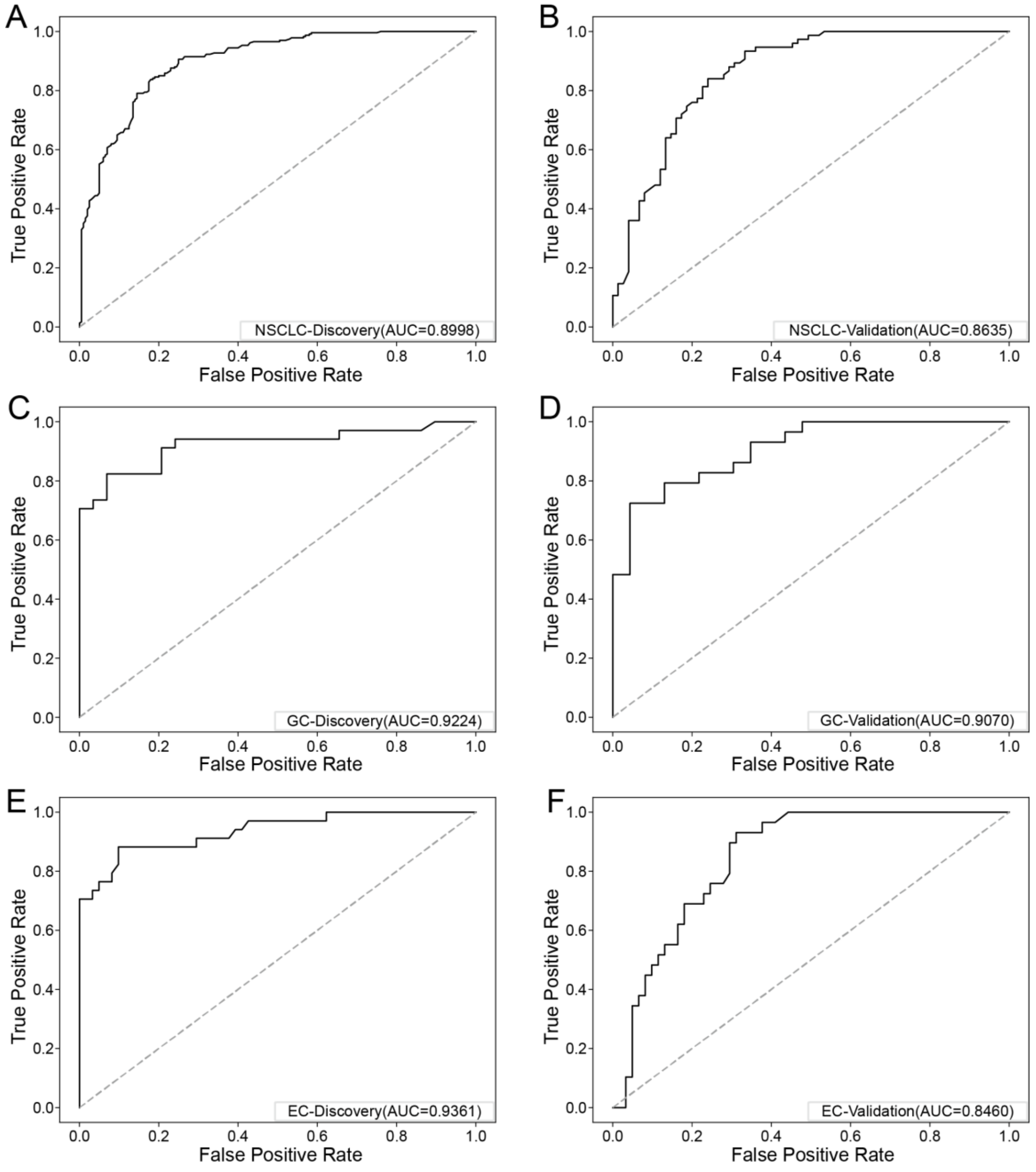

Figure S6. Area under the receiver operating characteristic curves show the classification performance of base model using N-glycome features in the discovery cohort and validation cohort, related to Figure 4. (A, B) Performance of base model in NSCLC group. **(C, D)** Performance of base model in GC group. **(E, F)**. Performance of base model in EC group. NSCLC, Non-small-cell lung cancer; GC, gastric cancer; EC, esophageal cancer.

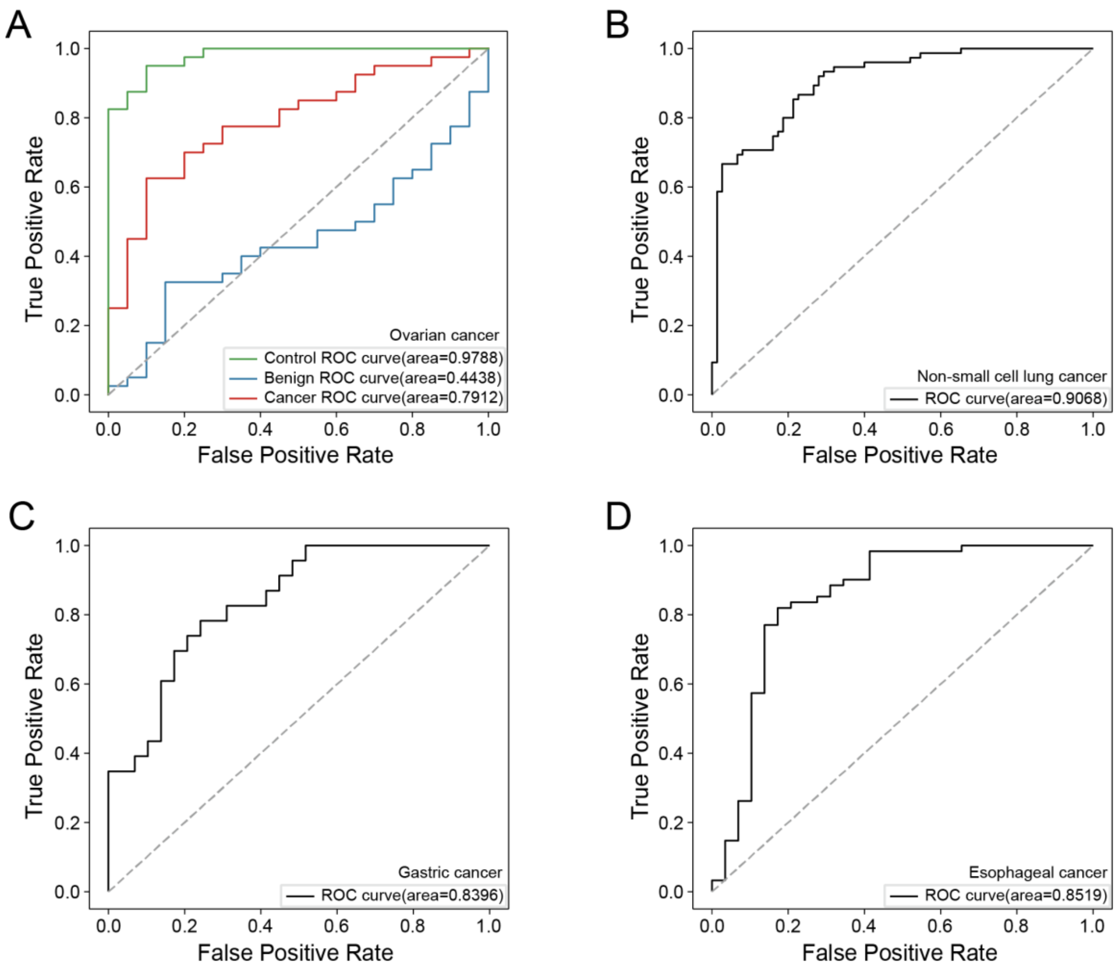

Figure S7. Area under the receiver operating characteristic curves show the classification performance of PLS-DA using N-glycome features in the validation cohort, related to Figure 5. (A), Performance of PLS-DA in ovarian cancer group. **(B)**, Performance of PLS-DA in non-small cell lung cancer group. **(C)**, Performance of PLS-DA in gastric cancer group **(D),** Performance of PLS-DA in esophageal cancer group.

Table S13. Formula for derived glycan traits calculation from detected glycan features, related to STAR Methods.

Glycan derived trait	Description	Formula
Man	Percentage of mannosylated structures in total serum glycans	(peak2+peak6+peak8+peak12+peak20+peak27) / (total peaks)
G0 Total	Percentage of agalactosylated structures in total serum glycans	(peak1+peak3+peak5+peak8+peak9+peak11+peak18) / (total peaks)
G1 Total	Percentage of mono-galactosylated structures in total serum glycans	(peak4+peak7+peak10+peak15+peak16+peak19+peak25) / (total peaks)
G2 Total	Percentage of di-galactosylated structures in total serum glycans	(peak17+peak24+peak26+peak32) / (total peaks)
G Total	Percentage of galactosylated structures in total serum glycans	(peak4+peak7+peak10+peak15+peak16+peak17+peak19+peak24+peak25+peak26+peak32+peak33) / (total peaks)
F Neutral	Percentage of neutral fucosylated structures in total serum glycans	(peak3+peak7+peak9+peak16+peak18+peak24+peak25+peak32) / (total peaks)
F Sialo	Percentage of sialylated structures of fucosylation in total serum glycans	(peak20+ peak29+ peak34+peak35+peak38+peak40+peak42+peak43+peak46+peak49+peak50+peak52+peak54) / (total peaks)
F Total	Percentage of structures with core-fucose in total serum glycans	(peak3+peak7+peak9+peak14+peak16+peak18+ peak21+peak24+peak25+peak29+peak32+peak34+peak35+peak38+peak40+peak42+peak43+peak46+peak49+peak50+peak52+peak54) / (total peaks)
B Neutral	Percentage of neutral bisected structures in total serum glycans	(peak11+peak18+peak25+peak26+peak32) / (total peaks)
B Sialo	Percentage of sialyted structures with bisecting GlcNAc in total serum glycans	(peak31+peak35+peak36+peak38+peak41+peak43 / (total peaks)
B Total	Percentage of bisected structures in total serum glycans	(peak11+peak18+peak25+peak26+peak31+peak32+peak35+peak36+peak38+peak41+peak43) / (total peaks)
S1 Total	Percentage of mono-sialylated structures in total serum glycans	(peak13+peak21+peak22+peak23+peak28+peak29+peak30+peak31+peak34+peak35+peak36+peak38+peak39+peak42+peak45) / (total peaks)
S2 Total	Percentage of di-sialylated structures in total serum glycans	(peak37+peak40+peak41+peak43+peak44+peak46+peak48+peak50) / (total peaks)
S3 total	Percentage of di-sialylated structures in total serum glycans	(peak47+peak49+peak51+peak52) / (total peaks)
S4 total	Percentage of di-sialylated structures in total serum glycans	(peak53+peak54) / (total peaks)
S Total	Percentage of sialylated structures in total serum glycans	(peak13+peak21+peak22+peak23+peak28+peak29+peak30+peak31+peak34+peak35+peak36+peak37+peak38+peak39+peak40+peak41+peak42+peak43+peak44

		+peak45+peak46+peak47+peak48+peak49+peak50 +peak51+peak52+ peak53+peak54) / (total peaks)
